# Acute-Phase-HDL Remodeling by Heparan Sulfate Generates a Novel Lipoprotein with Exceptional Cholesterol Efflux Activity from Macrophages

**DOI:** 10.1371/journal.pone.0003867

**Published:** 2008-12-05

**Authors:** Shui-Pang Tam, Robert Kisilevsky, John B. Ancsin

**Affiliations:** 1 Department of Pathology and Molecular Medicine, Queen's University, The Syl and Molly Apps Research Center, Kingston General Hospital, Kingston, Ontario, Canada; 2 Department of Biochemistry, Queen's University, The Syl and Molly Apps Research Center, Kingston General Hospital, Kingston, Ontario, Canada; University of Helsinki, Finland

## Abstract

During episodes of acute-inflammation high-density lipoproteins (HDL), the carrier of so-called good cholesterol, experiences a major change in apolipoprotein composition and becomes acute-phase HDL (AP-HDL). This altered, but physiologically important, HDL has an increased binding affinity for macrophages that is dependent on cell surface heparan sulfate (HS). While exploring the properties of AP-HDL∶HS interactions we discovered that HS caused significant remodeling of AP-HDL. The physical nature of this change in structure and its potential importance for cholesterol efflux from cholesterol-loaded macrophages was therefore investigated. In the presence of heparin, or HS, AP-HDL solutions at pH 5.2 became turbid within minutes. Analysis by centrifugation and gel electrophoresis indicated that AP-HDL was remodeled generating novel lipid poor particles composed only of apolipoprotein AI, which we designate β2. This remodeling is dependent on pH, glycosaminoglycan type, is promoted by Ca^2+^ and is independent of protease or lipase activity. Compared to HDL and AP-HDL, remodeled AP-HDL (S-HDL-SAA), containing β2 particles, demonstrated a 3-fold greater cholesterol efflux activity from cholesterol-loaded macrophage. Because the identified conditions causing this change in AP-HDL structure and function can exist physiologically at the surface of the macrophage, or in its endosomes, we postulate that AP-HDL contains latent functionalities that become apparent and active when it associates with macrophage cell surface/endosomal HS. In this way initial steps in the reverse cholesterol transport pathway are focused at sites of injury to mobilize cholesterol from macrophages that are actively participating in the phagocytosis of damaged membranes rich in cholesterol. The mechanism may also be of relevance to aspects of atherogenesis.

## Introduction

There is substantial evidence that high density lipoprotein (HDL) and its major apolipoprotein, apoA-I, can inhibit the initiation and progression of atherosclerosis [1]–[4]. The most widely accepted explanation for these effects is based on HDL's central role in reverse cholesterol transport (RCT), the transport of cholesterol from peripheral cells, like macrophages, to the liver for reuse or excretion as bile acids. The HDL fraction in plasma is composed of a polydisperse population of particles ranging from lipid poor small discoidal pre-β-HDL, to larger lipid-rich spherical HDL [5]. Pre-β-HDL collects cholesterol and phospholipids from parenchymal cells and in conjunction with lecithin cholesterol acyltransferase (LCAT), which transfers acyl groups from phosphatidylcholine to free cholesterol, the cholesterol is converted to an ester that is retained in the core of mature spherical HDL particles. ApoA-I is normally the most abundant protein (70–100%) on the various classes of HDL and plays a critical role in their functions. However, during the acute-phase response, the initial systemic reaction to inflammation, the plasma concentration of apoA-I declines substantially [6], [7] and is replaced by serum amyloid A (SAA), an acute-phase protein [8], [9].

SAA was identified in the early 1970's as the plasma protein responsible for forming tissue deposits called amyloid (AA-type) seen clinically in diseases with underlying persistent acute inflammation [10], [11]. Soon after its discovery, SAA was shown to be an acute-phase protein produced by the liver within hours of tissue injury regardless of cause. Its plasma concentration can increase a 1000-fold (1 µg/mg to 1 mg/ml) within 24 h and return to baseline levels over a 7–10 day period providing the tissue insult is short-lived [12], [13]. In plasma SAA is associated with HDL [13], [14] and during severe inflammation can contribute up to 80% of its apo-protein composition [15], [16]. The displaced apoA-I is rapidly cleared by the liver and kidneys [17], and together with a sharp decline in apoA-I gene expression during inflammation [18], [19] the apoA-I plasma half-life is reduced from 12 to 3.5 hr [6] with an overall 50–70% reduction in plasma concentration [19]–[22]. Since apoA-I plays such an important role in RCT the apolipoprotein compositional changes of HDL during inflammation have been viewed as being pro-atherogenic, a conclusion that may be premature.

An ancient protein conserved from echinoderms to humans (500–650 mya), SAA has no known homologue and until recently no clear function [23]. Several years ago we postulated that given SAA's association with HDL its function must be linked to that of HDL in RCT. The initial evidence for this idea centered on the observation that SAA decreases AP-HDL's binding affinity for hepatocytes by 2-fold while increasing its affinity for macrophages by up to 5-fold, and macrophages from inflamed mice have a 5-fold increase in binding sites for AP-HDL [24]–[26]. The binding to macrophage is dependent on the cell surface glycosaminoglycan, heparan sulfate (HS) [27] and likely involves a HS binding site in the C-terminal end of SAA [28]. Further investigations have shown that SAA also enhances macrophage uptake of AP-HDL [27], [29]–[31] and once inside the cell, SAA acts to mobilize cellular cholesterol for export [32]–[36]. Through the actions of two separate domains, SAA increases neutral cholesterol ester hydrolase (nCEH) and inhibits acyl-CoA cholesterol acyltransferase (ACAT) activities [34]–[36]. Intracellular cholesterol balance is thereby shifted from cholesterol ester to free cholesterol, the form which can be readily exported by ATP-binding cassette transport proteins (ABCA1, ABCG1), to extra-cellular HDL acceptors [37].

Although SAA's strong evolutionary conservation and regulated expression implies that its function must be of significant benefit to the host, it has been difficult to explain the survival advantage of promoting cholesterol mobilization and efflux during a period of apparent decline in apoA-I-based RCT potential. To address this question we now provide the first evidence that a rapid non-enzymatic mechanism exists for remodeling AP-HDL into apoA-I rich particles that have enhanced cholesterol-accepting activity complementing SAA's function.

## Results

### HS causes AP-HDL to aggregate at pH 5.2

**Figure 1:**
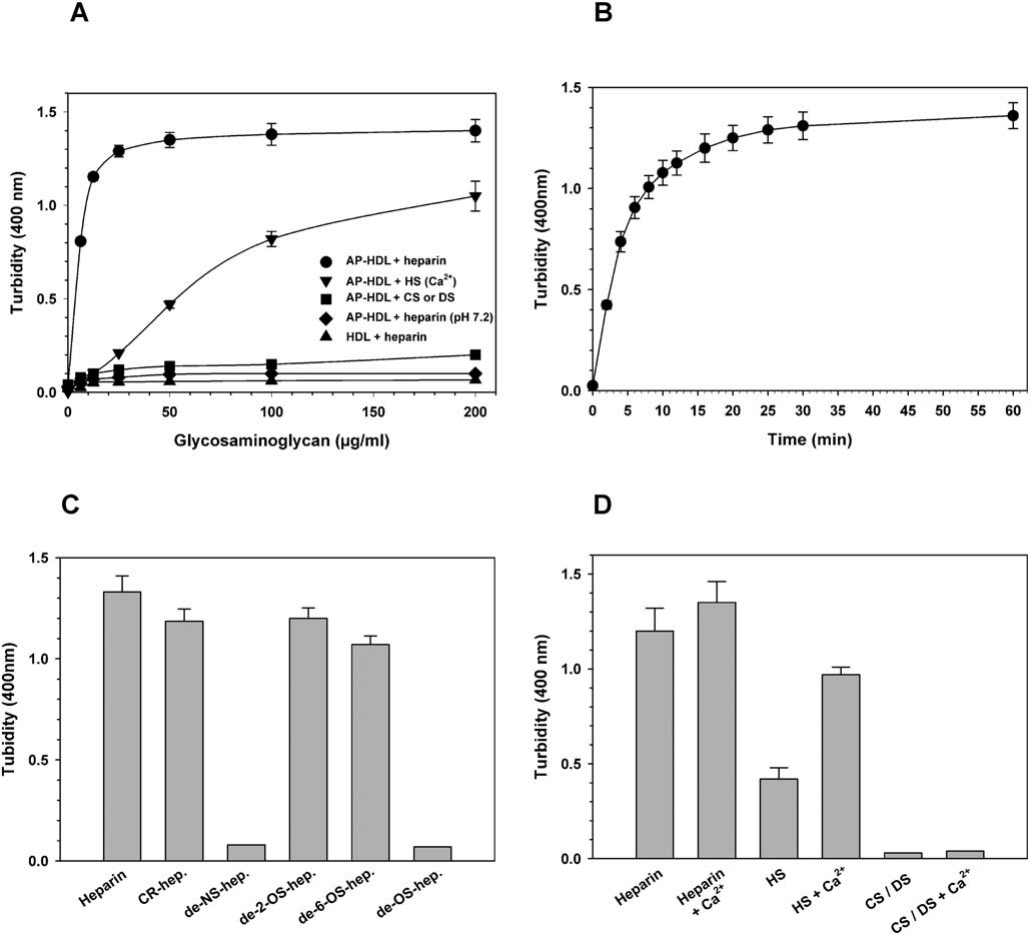
The aggregation of AP-HDL at mildly acid pH when incubated with heparin or HS. A) HDL or AP-HDL at pH 5.2 or 7.2 were incubated for 30 min with increasing concentrations of heparin, HS+1 mM Ca^2+^, CS (chondroitin sufate) or DS (dermatan sulfate). The absorbance at 400 nm was then plotted against glycosaminoglycan concentration. B) AP-HDL (1 mg/ml) at pH 5.2 in 125 mM NaCl, 25 mM Na acetate, was incubated with 0.2 mg/ml heparin and absorbance at 400 nm read at intervals to 60 min. C) AP-HDL (1 mg/ml) incubated for 20 min at 37°C with heparin, or different chemically modified heparins, at 0.1 mg/ml as described in Panel B; CR-hep. = carboxy-reduced heparin; de-NS-hep. = N-desulfated heparin; de-2-O-hep. = de-2-O-sulfated heparin; de-6-O-hep. = de-6-O-sulfated heparin; de-OS-hep. = de-2,6-O-sulfated heparin. D) Incubations, supplemented with 1 mM Ca^2+^, greatly increased the AP-HDL aggregation activity of HS. AP-HDL was incubated at pH 5.2 with heparin, HS, CS, DS with or without Ca^2+^, as in C). The results in C) and D) are the mean±SEM of 3 experiments. The results with CS and DS were virtually identical.

AP-HDL binding to macrophages requires cell surface HS-proteoglycans [27] and a HS binding site has been identified at the C-terminus of SAA [28]. Preliminary *in vitro* binding assays with AP-HDL revealed that incubation with commercially available HS, heparin (16 kDa) or heparin3000 (3 kDa) at mildly acidic pH (pH 5.2), caused AP-HDL to rapidly undergo a phase change and become visibly turbid (Figure 1A). This effect did not occur with HDL (Figure 1A) and was independent of protease or lipase activity. The results were the same in the presence and absence of protease inhibitors or EDTA (data not shown), and the effect of lipases are reported to take several hours to influence HDL structure as opposed to that seen with heparin, or HS, which occurs in minutes (Figure 1B).

The quantity of aggregate formed was saturable at sub-stoichiometric concentrations of heparin or HS (approx. 10 µM apoA-I to 4 µM heparin or HS) and reached 50% saturation in 5 min (Figure 1B). Centrifugation (10,000×g, 3 min.) produced supernatant fractions (S-HDL-SAA) clear of aggregates and pelleted aggregates (A-HDL-SAA) with a protein content proportional to the degree of turbidity, saturating at aproximately 35% of total AP-HDL protein content (data not shown). Aggregation was not produced with other glycosaminoglycans, such as chondroitin or dermatan sulfates, indicating a degree of specificity for HS and that charge is not the only parameter of importance in this reaction. Furthermore, aggregation was dependent on N-sulfation of the glucosaminyl residues of heparin (Figure 1C). For HS the addition 1 mM Ca^2+^ increased turbidity a further 2-fold (Figure 1D). Mildly acidic-pH alone did not cause any disruption in AP-HDL structure as judged by gel filtration (not shown) and in agreement with recent reports that HDL is a highly stable structure [38], [39].

### HS remodels AP-HDL into lipid-poor apoA-I rich particles

**Figure 2:**
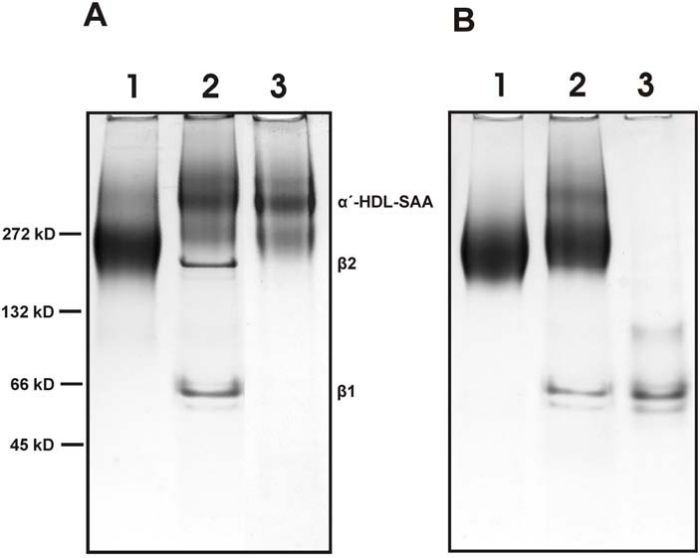
Non-denaturing polyacylamide gel electrophoresis (N-PAGE) of normal and heparin treated HDLs. AP-HDL, S-HDL-SAA and HDL were resolved by N-PAGE using 4–20% gradient gels with a continuous buffer system consisting of 0.1 M glycine-Tris-HCl, pH 8.6. Samples (30 µg) were loaded onto the gel and electrophoresis carried out at 4°C for 6 h at 150 volts. Bands were detected by staining with Coomassie Blue R250. A), lane 1, AP-HDL, lane 2, S-HDL-SAA, lane 3, float sample after re-centrifugation of S-HDL-SAA at d = 1.25 g/ml. Molecular weight markers are indicated on the left; B), lane 1, HDL, lane 2, S-HDL, lane 3, delipidated apoA-I.

Electrophoresis of the S-HDL-SAA fraction using non-denaturing PAGE revealed several new components not apparent in untreated AP-HDL. One component was ∼60 kDa (β1) and the other ∼180 kDa (β2) (Figure 2A, lane 2). Analysis of these two bands by SDS-PAGE and tryptic mass spectrometry revealed that the protein composition of both β1 and β2 was entirely apoA-I. Residual SAA and other apo -proteins remained with the α-HDL band. Re-centrifugation of the S-HDL-SAA fraction at a density of 1.25 g/ml resulted in the preferential loss of the β species confirming their lipid poor compositions (Figure 2A, lane 3). No increase in turbidity or obvious aggregation occurred when HDL was incubated with heparin, although some β1 was detected on PAGE (Figure 2B, lane 2). ApoA-I purified from HDL-SAA co-migrated with β1 indicating that it is composed of pure delipidated apoA-I dissociated from HDL (Figure 2B, lane 3). Delipidated apoA-I's (28.2 kDal) tendency to form dimers has been previously reported [40], [41].

### β2-particles have exceptional cholesterol accepting properties

**Figure 3:**
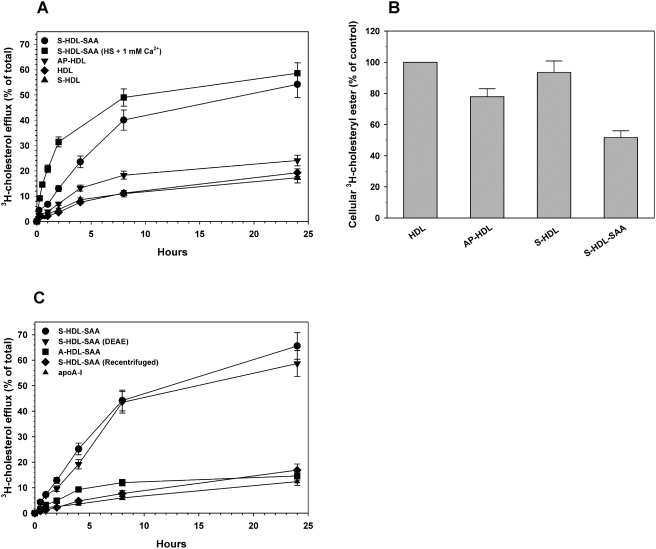
Cholesterol efflux from J774 monocytes incubated with different lipoproteins. Monolayer cultures of J774 monocytes were loaded with cholesterol (22.7 µg/well) and [^3^H]-cholesterol (0.45 µCi) overnight, washed twice with DMEM in 0.2% BSA (1 ml) and once with DMEM alone. Cholesterol-loaded cells were then incubated with different lipoprotein preparations (50 µg/ml) in 1 ml DMEM, 0.2% BSA; A) S-HDL-SAA prepared with heparin; S-HDL-SAA prepared with HS+1 mM Ca2+; A-HDL-SAA; AP-HDL; HDL; S-HDL prepared with heparin. B) Lipid was extracted from cell and analyzed by thin-layer chromatography as described previously [35] to determine the amount of [3H]-cholestryl ester remaining in the cells relative to cholesterol-loaded cells. C) S-HDL-SAA; S-HDL-SAA preincubated with DEAE-Sepharose to remove any heparin; S-HDL-SAA recentrifuged at 1.25 g/ml to remove lipid poor species, A-HDL-SAA and delipidated apoA-I. Aliquots of medium were taken at the time intervals shown and the quantity of [^3^H]-cholesterol exported from the cells determined and expressed as a percentage of total [^3^H]-cholesterol in loaded cells before incubations.

The S-HDL-SAA fraction is an exceptional acceptor of cholesterol from cholesterol-laden macrophages. Whether generated with heparin, or HS (+1 mM Ca^2+^), the resulting S-HDL-SAA had a 2.5 to 3-fold greater cholesterol accepting activity relative to AP-HDL (Figure 3A). The bulk of the exported cholesterol was derived from de-esterified cellular cholesterol esters (Figure 3B). Analysis of S-HDL-SAA cholesterol content by thin layer chromatography confirmed that only free-cholesterol was accepted from cholesterol-loaded cells (data not shown). The presence of heparin in the S-HDL-SAA fraction did not contribute to this activity since its removal by ion exchange chromatography had no effect on the proportion, or rate of cholesterol removed from the cells (Figure 3C). Depletion of the β-components from the S-HDL-SAA fraction clearly abolished its ability to promote cholesterol efflux (Figure 3C). Heparin treated HDL also produced some β1 but no increase in activity was evident and pure apoA-I demonstrated poor efflux activity. These data point to the β2 species as the one playing a significant role in macrophage cholesterol efflux.

## Discussion

When macrophages take up cholesterol any excess not required for membrane homeostasis or other functions is esterified by ACAT and stored in ester form. These cholesterol-ester loads can be significant for macrophages transforming them into “foam cell”, one of the earliest cellular indicators of atherogenesis in arterial disease. The first step in the mobilization and export of excess stored cholesterol via the RCT pathway is the de-esterification of cholesterol-esters accomplished by nCEH. The resulting free cholesterol moves through the cell membrane either by passive diffusion or active transport by ABC transport proteins (ie. ABCA1, ABCG1) for collection and transport by apoA-I-rich lipoproteins.

Recent studies have demonstrated that the acute-phase protein SAA contains two domains one of which inhibits ACAT activity and the other enhances nCEH activity thereby substantially shifting the balance of cellular cholesterol to that of free cholesterol available for efflux [34]–[36]. Neither of these two activities is affected by apoA-I [33]–[35] and since β1 and β2 described herein consist entirely of apoA-I it is not likely that they directly promote the de-esterification of stored cholesterol esters. Furthermore, HDL treated with heparin generated low levels of β1, but not β2. Although we cannot completely rule out the possibility that β1 contributed to the increase in cholesterol efflux observed for S-HDL-SAA, its presence did not improve the efflux activity for HDL. Also, the electrophoretic mobility of β1 suggests that it is composed of lipid free apoA-I, which was not an efficient acceptor of cholesterol from macrophages in our assays (Figures 3A and 3B). Conversely, SAA, or its active peptide domains, can influence ACAT and nCEH activities even in the absence of cholesterol export but requires a cholesterol acceptor, such as HDL, for rapid and efficient macrophage cholesterol efflux [33], [35]. Efflux promoted by SAA was demonstrated to be dependent on the activity of ABCA1 and/or ABCG1 [34]. Moreover, we have shown previously that the *in vitro* effect of AP-HDL (i.e. HDL-SAA) on macrophage cholesterol efflux can be demonstrated *in vivo* [34]–[36], [42]. Cholesterol-laden macrophages when injected intravenously into normal mice and allowed to acclimatize themselves for 24 h rapidly export their cholesterol in response to HDL-SAA, or SAA's active domains, in the same manner as they do *in vitro*. Our present data argue that apoA-I in the form of the β2 component is an exceptionally efficient cholesterol acceptor of exportable cholesterol. We therefore postulate that AP-HDL contains latent functionalities that become active when it associates with macrophage cell surface/endosomal HS at least at sites of injury and possibly elsewhere. The α′-component of S-HDL-SAA contains SAA, which by virtue of its two active domains drives the de-esterification of cholesterol esters and prevents its re-esterification. The β2-component is the efficient extracellular cholesterol acceptor that gets targeted to macrophages by virtue of AP-HDL's increased affinity and the macrophages increased number of binding sites for AP-HDL.

The data presented herein suggests that AP-HDL on binding to HS under mildly acidic pH associated with normal membrane electrostatics [43], [44] or endosomal compartments, can rapidly be remodeled generating novel apoA-I-rich particles that function physiologically to mobilize cholesterol from cholesterol-laden macrophages at sites of tissue injury. The micro-environmental conditions favoring HS-based remodeling of AP-HDL may occur at sites of injury, and potentially elsewhere where macrophages are actively scavenging damaged cell membranes. Macrophage are exceptional at endosomal acidification reaching pH 5.0–5.5 within 10 min of endocytosis [45]. Furthermore, sites of tissue injury can become acidic due to reduced blood supply leading to hypoxia which forces cells to switch to anaerobic glycolysis resulting in lactic acidosis.

We believe that the described mechanism for remodeling AP-HDL explains why AP-HDL is consistently more effective than HDL as a promoter of macrophage cholesterol efflux [34] (Figure 3A). Allowing AP-HDL to interact with heparin/HS *in vitro*, likely replicates, under optimum conditions, the physiological process that takes place at the surface of the macrophage at sites of injury. This re-modeling of AP-HDL takes place in minutes, does not require lipases and is a natural consequence AP-HDL's interaction with HS likely mediated through a HS-binding site identified on SAA [28].

As we have argued previously [8] the actions of SAA may help conserve cholesterol pools during severe injury. The biosynthesis of cholesterol is a significant metabolic investment requiring 30-enzymatic steps. SAA's influence would decrease cholesterol loss, allow its recycling and compensate for any injury related decline of cholesterol biosynthesis/availability that is necessary for post-injury tissue repair. SAA's activities may also improve the cellular performance of macrophages at sites of injury by limiting foam cell formation. Evidence for this latter point has recently been provided using an experimental atherogenesis model, where the development of aortic lipid lesions in apo-E-/- mice was effectively prevented and even reversed by intravenous injections of liposomal formulations of synthetic peptides corresponding to SAA's two functional domains [36].

## Methods

### Chemicals

All chemicals were reagent grade and purchased from Fisher Scientific (Nepean, Ontario), Sigma (St. Louis, MO), ICN (Aurora, OH), or BioRad (Hercules, CA). Dulbecco's modified Eagle's medium (DMEM) and FBS were purchased from Life Technologies (Burlington, Ontario). Radiolabeled [1,2,6,7-^3^H(N)] cholesterol (82 Ci/mmol) was obtained from DuPont NEN (Boston, MA). Glycosaminoglycans and SIGMAFAST™ Protease Inhibitor were purchased from Sigma (St. Louis, MO).

### Purification of HDL, AP-HDL and apoA-I

To increase plasma concentrations of SAA in CD1 mice an acute-inflammatory state was induced by a subcutaneous injection of 0.5 ml 2% (w/v) AgNO_3_ [46]. After 24 h, mice were sacrificed by CO_2_ narcosis and exsanguinated by cardiac puncture preventing clotting with EDTA. High density lipoprotein (HDL) containing SAA (AP-HDL) was isolated from inflamed plasma by sequential density flotation [47]. The density of the plasma was adjusted to 1.063 g/ml with NaBr and centrifuged at 175,000 g for 18 h in a 70.1Ti rotor (Beckman) at 10°C. The top layer containing VLDL/LDL was removed and discarded. The pooled infranatants were adjusted to a density of 1.21 g/ml and re-centrifuged at 250,000 g for 24–36 h at 10°C. The top layer (AP-HDL) was aspirated, pooled, and dialysed against 20 mM Tris-HCl, 0.15 M NaCl, 0.1% (w/v) EDTA, pH 7.5, for 18 h. Lipoproteins were assessed by reverse phase-high pressure liquid chromatography on a C18-Vydac column as previously described [28]. The eluant containing apoA-I was collected, lyophilized and stored at −20°C.

### AP-HDL and HDL aggregation

Lipoproteins were applied to a Sephadex G-50 column (1×10 cm) equilibrated in 0.15 M NaCl. Void fractions were collected and adjusted to 2 mg/ml protein, then stored on ice. HDL or AP-HDL were diluted to a final concentration of 1 mg/ml in 25 mM sodium acetate, 125 mM NaCl, pH 5.2 or pH 7.2 containing increasing concentrations of heparin, HS, chondroitin sulfate (CS) or dermatan sulfate (DS). In some experiments a broad spectrum protease inhibitor (Sigma) was included to judge the involvement of proteolysis. Upon mixing tubes were incubated in a water bath at 37°C for 0–60 min. Tubes were re-mixed and absorbance read at 400 nm in a quartz cuvette (path length 1 cm). Aggregates were collected by centrifugation at 10,000×g×3 min. producing a pellet and clarified supernatant. Pellet was washed once, re-suspended in buffer and both pellet (A-HDL-SAA) and supernatant fractions (S-HDL-SAA) were dialysed against 20 mM HEPES, 150 mM NaCl, pH 7.2, at 4°C. S-HDL-SAA was concentrated to 1–2 mg/ml by ultrafiltration using a Centricon (YM10) concentrator and stored on ice until use. Heparin was purified from some S-HDL-SAA samples by anion exchange chromatography on DEAE-Sepharose-CL 4B column [48]. After centrifugation to pellet the aggregates the supernatant containing S-HDL-SAA fraction was applied to the column pre-equibrated with 25 mM sodium acetate, 125 mM NaCl, pH 5.2 and the flow through containing the S-HDL-SAA was collected with heparin remaining bound to the column.

### Cell culture and cholesterol loading

J774 macrophages (American Type Tissue Collection, Manassas, VA) were cultured in 6-well tissue culture plates at one million cells per well and grown to 90% confluence in 2 ml DMEM supplemented with 10% FBS. The medium was changed three times a week. Cholesterol loading of cells was carried out using β-methyl-cyclodextrin (MCD). A stock solution of 30 mM MCD in phosphate-buffered saline (PBS) was prepared. Two ml of MCD (30 mM) was mixed with 0.2 ml of cholesterol dissolved in ethanol (5 mg/ml) and 20 µl of ^3^H-cholesterol (20 µCi). The mixture was incubated at 37°C for 1 h. For cholesterol loading and labeling of the J774 cells, 50 µl of the MCD-cholesterol complex was added to 1 ml of DMEM in 0.2% BSA overnight. After incubation, the cells were washed twice with DMEM in 0.2% BSA (1 ml) and once with DMEM alone prior to cholesterol efflux studies that were carried out in DMEM-BSA.
